# Time

**Published:** 1891-07

**Authors:** 


### TIME.

Unfathomable Sea! whose waves are years,
  Ocean of Time, whose waters of deep woe
Are brackish with the salt of human tears!
  Thou shoreless flood, which in thy ebb and flow
    Claspest the limits of mortality!
And sick of prey, yet howling on for more,
Vomitest thy wrecks on its inhospitable shore,
  Treacherous in calm, and terrible in storm,
    Who shall put forth on thee,
    Unfathomable Sea?—SHELLY.

